# Nanocomposite-Based Dual Electrochemical Immunosensor for Simultaneous Detection of Intestinal Barrier Biomarkers: Intestinal Fatty Acid Binding Protein and Fecal Calprotectin

**DOI:** 10.3390/bios16040199

**Published:** 2026-04-01

**Authors:** Lorena García-Rodrigo, Claudia Ramos-López, Esther Sánchez-Tirado, Lourdes Agüí, Araceli González-Cortés

**Affiliations:** Department of Analytical Chemistry, Faculty of Chemical Sciences, Universidad Complutense of Madrid, 28040 Madrid, Spain; lorega05@ucm.es (L.G.-R.); claura07@ucm.es (C.R.-L.); malagui@ucm.es (L.A.)

**Keywords:** iFABP, FC, V_2_O_5_/MWCNTs nanocomposite, SPdCEs, fecal samples

## Abstract

Disruption of the intestinal barrier is a hallmark of inflammatory bowel disease (IBD) and drives both epithelial injury and neutrophil-mediated inflammation, yet rapid, multiplexed assessment of these processes remains an unmet clinical need. Intestinal fatty acid binding protein (iFABP) and fecal calprotectin (FC) provide complementary insights into barrier integrity and mucosal inflammation, but conventional ELISA-based assays are time-consuming, low-throughput, and require large sample volumes. Here, we introduce a dual electrochemical sandwich immunosensor enabling simultaneous quantification of iFABP and FC on screen-printed dual carbon electrodes (SPdCEs). Capture antibodies were immobilized via electrografting of *p*-aminobenzoic acid diazonium salt, while a V_2_O_5_/MWCNTs-HRP–streptavidin nanocomposite amplified the electrocatalytic reduction in hydrogen peroxide, enhancing sensitivity. The platform achieved detection limits of 0.01 pg mL^−1^ (iFABP) and 1 pg mL^−1^ (FC) with a total assay time of 1 h 20 min and sample volume of just 5 μL, outperforming conventional ELISA in speed and efficiency. High repeatability, reproducibility, and accurate recovery in enriched fecal samples confirmed analytical robustness. By integrating multiplexed detection, nanostructured signal amplification, and robust electrode engineering, this immunosensor provides a rapid, sensitive, and low-volume platform for point-of-care and decentralized monitoring of IBD, enabling timely clinical decision-making and longitudinal patient management.

## 1. Introduction

Inflammatory bowel disease (IBD) is a chronic disorder affecting a significant portion of the global population and presenting major clinical and economic challenges [1,2]. Its two main forms, Crohn’s disease (CD) and ulcerative colitis (UC), differ in pathological patterns, with UC mostly confined to the colon and CD affecting multiple regions of the gastrointestinal tract. The interplay between the intestinal barrier, immune responses, environmental factors, and microbiota underlies disease onset and progression, yet the mechanisms remain incompletely understood [3]. Maintaining epithelial integrity is essential, as its disruption can trigger and amplify inflammation.

Non-invasive biomarkers such as intestinal fatty acid-binding protein (iFABP) and fecal calprotectin (FC) offer complementary insights into disease activity [4]. iFABP reflects epithelial damage, while FC reports neutrophil-mediated mucosal inflammation. Clinically, iFABP is typically present at low circulating levels in healthy individuals (generally < 300 pg mL^−1^), while markedly increased concentrations are associated with epithelial damage and increased in patients with intestinal injury, including IBD-related conditions [5,6]. In parallel, fecal calprotectin levels below 50 µg g^−1^ are considered indicative of normal intestinal homeostasis, whereas concentrations above 150–200 µg g^−1^ are strongly linked to active intestinal inflammation and disease activity in IBD patients [7,8,9]. Combined detection enables differentiation between epithelial-driven injury and immune-mediated responses, providing a more comprehensive assessment of IBD pathophysiology [7,10].

Conventional ELISA assays, though sensitive, require long processing times and centralized laboratories. Recent efforts focus on electrochemical biosensors for rapid, portable, and cost-effective biomarker detection [4,7,11,12]. Hybrid nanomaterials, particularly vanadium pentoxide (V_2_O_5_) combined with multiwalled carbon nanotubes (MWCNTs), enhance signal transduction, catalytic activity, and analytical performance, supporting sensitive and multiplexed detection [13,14,15,16,17].

Here, we report a dual electrochemical sandwich immunosensor for simultaneous quantification of iFABP and FC using screen-printed dual carbon electrodes. Capture antibodies were immobilized via electrografting [18], followed by sandwich complex formation with biotinylated secondary antibodies. Signal amplification was achieved with a V_2_O_5_/MWCNTs/HRP-streptavidin nanocomposite, enabling rapid and selective detection in fortified fecal samples. This platform provides a practical, point-of-care approach for monitoring IBD, potentially supporting timely clinical decisions and decentralized patient care.

## 2. Materials and Methods

### 2.1. Instrumentation

Electrochemical measurements were performed at room temperature. Amperometric experiments were carried out using a CH1 1030B potentiostat (Chemical Instruments, Inc., Austin, TX, USA) controlled via CH1 1030B software. Electrochemical impedance spectroscopy (EIS) and related analyses were conducted using a μAutolab Type III potentiostat (Ecochemie, FRA2 software, Utrecht, The Netherlands) and the Nova 2.1.5 system (Metrohm Autolab B.V., Utrecht, The Netherlands). Single (SPCE, DRP-110, 4 mm diameter, Llanera (Asturias), Spain) and dual carbon screen-printed electrodes (SPdCE, X1110 DRP, two elliptical working electrodes, total area 4.7 mm^2^, Llanera (Asturias), Spain) were employed, each including a carbon counter electrode and silver pseudo-reference electrode. Measurements were performed in stirred 10 mL glass cells. Morphology and chemical composition of nanomaterials were characterized using SEM, TEM, and Raman spectroscopy. Supporting equipment included a pH meter, ultrasonic bath, homogenizer, and centrifuge.

### 2.2. Chemicals and Reagents

Anti-iFABP capture antibody, human iFABP standard, and biotinylated anti-iFABP detection antibody were obtained from the FABP2/iFABP DuoSet ELISA kit (R&D Systems, Minneapolis, MN, USA, Cat. No. DY3078). Anti-FC capture antibody, human FC standard, and biotinylated anti-FC detection antibody were sourced from the Human S100A8/S100A9 Heterodimer ELISA Kit—Quantikine (R&D Systems, Cat. No. DY8226). Horseradish peroxidase–streptavidin (HRP-Strep) was purchased from Pierce Thermo Scientific (Rockford, IL, USA).

Cross-linking and electrochemical reagents, including *N*-hydroxysulfosuccinimide (sulfo-NHS), *N*-(3-dimethylaminopropyl)-*N*^′^-ethylcarbodiimide (EDC), hydroquinone (HQ), hydrogen peroxide (H_2_O_2_, 30% *v*/*v*), *p*-aminobenzoic acid (*p*ABA, ≥99%), potassium ferricyanide (III) (K_3_[Fe(CN)_6_], 99.0%), potassium ferrocyanide (II) (K_4_[Fe(CN)_6_], 98.5–102.0%), vanadium pentoxide (V_2_O_5_, 99%), and sodium nitrite (NaNO_2_, ≥97%), were purchased from Merck. Buffer salts, including sodium monobasic phosphate (NaH_2_PO_4_, ≥99%), sodium dibasic phosphate (Na_2_HPO_4_, ≥99%), and potassium chloride (KCl, 99–100.5%), which were supplied by Scharlab. Multi-walled carbon nanotubes (MWCNTs, ϕ 30 ± 15 nm, 95% purity) were obtained from NanoLab (Brighton, MA, USA).

Buffer solutions were prepared as follows: 25 mM MES (pH 5.0) from 2-(N-morpholino)ethanesulfonic acid (Gerbu, Heidelberg, Germany), 50 mM and 100 mM phosphate buffers (PBs) at pH 6.0 from Na_2_HPO_4_ and NaH_2_PO_4_, and phosphate-buffered saline (PBS, pH 7.4) consisting of 100 mM sodium phosphate with 2.0 g NaCl and 50.25 mg KCl in 250 mL deionized water. All solutions were prepared using high-purity deionized water (18.2 MΩ·cm at 25 °C; TOC < 10 µg L^−1^) from a Millipore Milli-Q system.

### 2.3. Synthesis of V_2_O_5_/MWCNTs Nanocomposite

The V_2_O_5_/MWCNT nanocomposite was prepared following a multi-step procedure adapted from Han et al. [19]. Briefly, 335 mg of MWCNTs were functionalized via acid treatment (H_2_SO_4_/HNO_3_, 3:1 *v*/*v*) at 40 °C for 2 h under stirring, then washed thoroughly with water. In parallel, 102 mg of V_2_O_5_ was dissolved gradually in 8 mL of 30% H_2_O_2_. Functionalized MWCNTs were added to the solution and stirred for 8 h, then allowed to stand for 24 h. The suspension was transferred to a Teflon-lined autoclave and hydrothermally treated at 180 °C for 72 h. The product was washed with water and ethanol until the supernatant was clear, and dried under vacuum at 80 °C.

### 2.4. Preparation of V_2_O_5_/MWCNTs-HRP–Streptavidin Bioconjugate

A total of 1 mg of V_2_O_5_/MWCNTs nanocomposite was dispersed in 1 mL MES buffer. Carboxyl groups were activated with EDC and sulfo-NHS, followed by addition of 1 μL of HRP-streptavidin (500 U/mL). The mixture was incubated with gentle stirring to allow covalent attachment. The bioconjugate was washed five times with PBS by centrifugation (6000× *g*, 5 min) and resuspended in fresh PBS for further use.

### 2.5. Construction of the Dual Electrochemical Immunosensor

SPdCE surfaces were functionalized by electrochemical grafting of *p*ABA diazonium salt, generated in situ from 20 mg *p*ABA in 2 mL 1 M HCl with 2 mM NaNO_2_ under ice bath conditions. Ten cyclic voltammetry scans (0.0 to –1.0 V vs. Ag pseudo-reference, 200 mV s^−1^) were performed. Carboxyl groups were activated using 10 μL of 0.1 M EDC/sulfo-NHS in MES buffer (pH 5.0) for 30 min. Capture antibodies (anti-iFABP, 15 μg·mL^−1^; anti-FC, 10 μg·mL^−1^) were deposited onto the respective electrodes and incubated for 30 min. Unreacted sites were blocked with BSA (6% W1, 3% W2) for 30 min.

**Figure 1:**
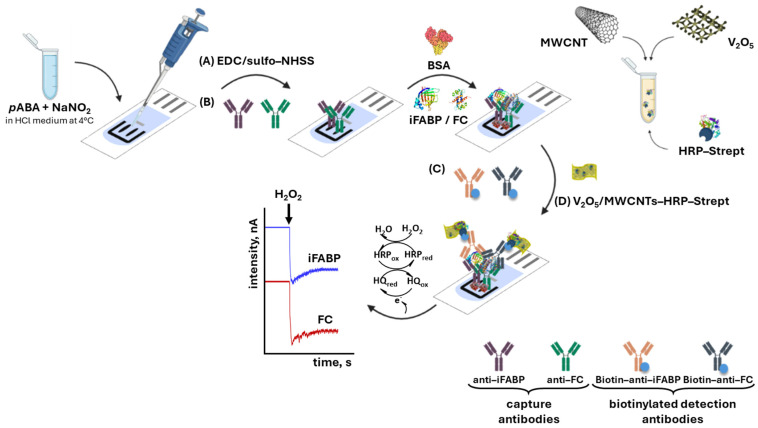
Schematic illustration of the stepwise fabrication and operating principle of the electrochemical dual immunosensor for the simultaneous determination of iFABP and FC. The procedure includes (i) electrochemical grafting of *p*-aminobenzoic acid diazonium salt onto SPdCEs to introduce carboxylic groups, followed by EDC/sulfo-NHS activation; (ii) covalent immobilization of capture antibodies (anti-iFABP and anti-FC); (iii) surface blocking with BSA to minimize nonspecific adsorption; (iv) formation of the sandwich immunocomplex through antigen binding and subsequent incubation with biotinylated detection antibodies; and (v) signal amplification using the V_2_O_5_/MWCNTs-HRP–streptavidin nanocomposite via biotin–streptavidin interaction. The analytical signal is generated by the catalytic reduction in H_2_O_2_, combining the peroxidase-like activity of V_2_O_5_ and the enzymatic activity of HRP, and recorded amperometrically for each biomarker.

For detection, 5 μL of standards or samples were applied for 15 min, followed by 30 min incubation with biotinylated detection antibodies (0.5 μg/mL). Signal amplification was performed by adding 5 μL of 1:10 diluted V_2_O_5_/MWCNTs-HRP–Streptavidin composite for 20 min. This nanocomposite binds to the biotinylated detection antibody through the streptavidin–biotin interaction, enabling combined nanozyme and enzymatic signal amplification. All steps were carried out at room temperature in a humid environment (Figure 1).

### 2.6. Electrochemical Characterization and Measurement

Cyclic voltammetry and EIS were performed in 0.1 M KCl containing 5 mM [Fe(CN)_6_]^3−/4−^. CV scans ranged from −0.1 to 0.6 V vs. Ag/AgCl. EIS spectra were collected from 0.1 to 10^5^ Hz with 0.01 V AC amplitude under open-circuit conditions. Amperometric measurements were conducted in 50 mM phosphate buffer (pH 6.0) with hydroquinone (100 mM). A potential of −0.20 V vs. Ag pseudo-reference was applied, and cathodic current changes after addition of 50 μL of 100 mM H_2_O_2_ were monitored to steady state (~100 s). Signals represent the mean of three independent replicates ±3 × SD (α = 0.05).

### 2.7. Testing Fecal Samples

Fecal samples from healthy volunteers were enriched with clinically relevant concentrations of iFABP and FC [4,16,17]. Samples were weighed into a dedicated container (OC-Auto Sampling Bottle 3, Ref. V-PZ25, Eiken Chemical Co. Ltd., Tokyo, Japan) and dispersed in 2 mL of HEPES buffer through vigorous manual mixing. For electrochemical assays, 5 μL aliquots of the resulting suspension were applied to the sensor. All procedures adhered to institutional ethical guidelines and regulatory standards.

## 3. Results

### 3.1. Morphological Characterization Studies

**Figure 2:**
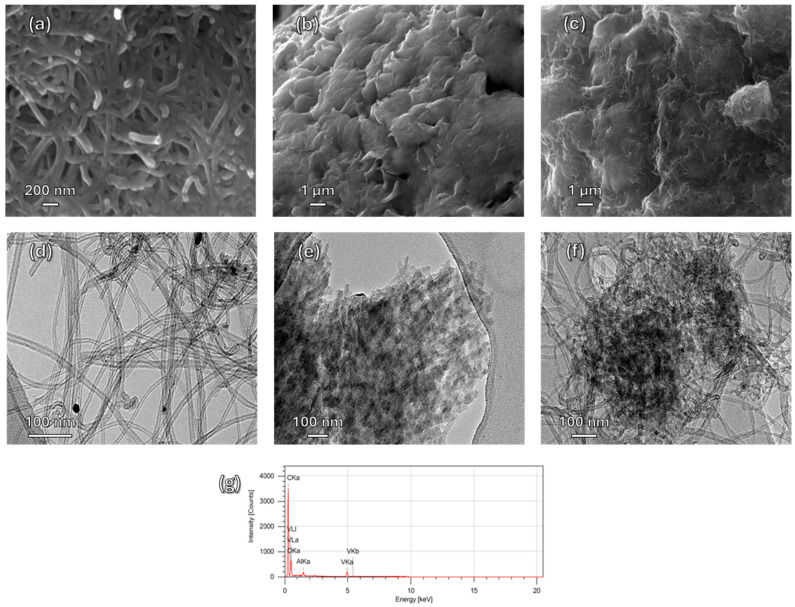
SEM micrograph of (**a**) MWCNTs, (**b**) V_2_O_5_, (**c**) V_2_O_5_/MWCNTs, showing the morphological features and surface structure of each material; TEM micrograph of (**d**) MWCNTs, (**e**) V_2_O_5_, (**f**) V_2_O_5_/MWCNTs illustrating the nanostructure and distribution of V_2_O_5_ within the carbon nanotube network, and (**g**) EDX spectrum of V_2_O_5_/MWCNTs confirming the elemental composition of the composite through the presence of characteristic signals of V, O, and C. Scale bars: (**a**) 200 nm; (**b**,**c**) 1 µm; (**d**–**f**) 100 nm.

The structural features of MWCNTs, V_2_O_5_, and the V_2_O_5_/MWCNTs hybrid were examined using SEM and TEM. MWCNTs displayed well-defined cylindrical tubes with uniform distribution (Figure 2a), whereas V_2_O_5_ appeared as irregular, porous flakes (Figure 2b). In the composite, V_2_O_5_ flakes were dispersed across the nanotube network, creating a rougher surface compared to pristine MWCNTs (Figure 2c). TEM confirmed that the nanotubes retained their integrity and that V_2_O_5_ flakes were attached along the external walls, indicating effective formation of the hybrid structure (Figure 2f) [5,20]. EDX analysis (Figure 2g) verified the presence of C, O, and V elements, confirming successful incorporation of V_2_O_5_.

Raman spectroscopy (Figure S1) revealed characteristic bands for each material. MWCNTs showed D (1350 cm^−1^) and G (1590 cm^−1^) bands, while V_2_O_5_ exhibited vibrational modes at 155, 283, 410, 530, 698, and 995 cm^−1^, consistent with its orthorhombic structure. In the V_2_O_5_/MWCNT composite, the terminal V=O stretching at 995 cm^−1^ disappeared, suggesting oxide reorganization and strong interaction with the nanotube framework [21,22,23,24].

### 3.2. Peroxidase-Mimetic Activity of V_2_O_5_/MWCNTs of Nanocomposite

The catalytic activity of the V_2_O_5_/MWCNTs hybrid was evaluated using the TMB/H_2_O_2_ chromogenic reaction. Incremental addition of the nanocomposite produced progressive blue coloration, indicating hydrogen peroxide activation in a concentration-dependent manner (Figure S2). Controls confirmed that MWCNTs alone had negligible catalytic effect, whereas V_2_O_5_ retained peroxidase-like activity. Integration with MWCNTs preserved redox activity while facilitating electron transfer.

These results indicate that the peroxidase-mimetic behavior is mainly associated with the V_2_O_5_ phase, in agreement with previous reports describing vanadium oxide nanozymes as efficient catalysts for H_2_O_2_ activation [17,19]. In this context, V_2_O_5_ is considered the main component responsible for the peroxidase-like activity of the composite, while MWCNTs mainly act as a conductive support that improves electron transfer and favors the effective dispersion and accessibility of the catalytic sites. Therefore, the enhanced response of the hybrid material can be attributed to a synergistic effect between the intrinsic catalytic activity of V_2_O_5_ and the high conductivity and large surface area of the MWCNT network.

**Figure 3:**
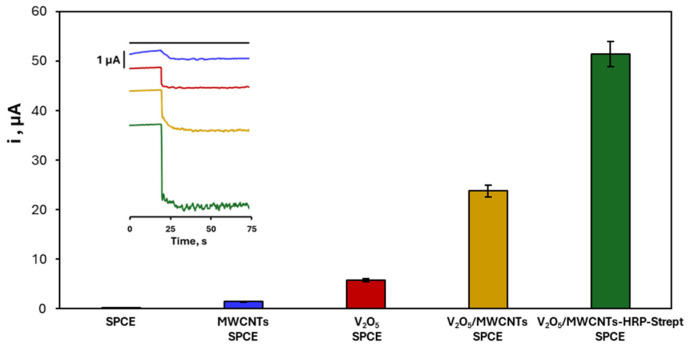
Comparison of the catalytic current responses obtained with different modified screen-printed carbon electrodes (SPCEs): bare SPCE, MWCNTs/SPCE, V_2_O_5_/SPCE, V_2_O_5_/MWCNTs/SPCE, and V_2_O_5_/MWCNTs-HRP-Strept/SPCE. The bars represent the steady-state current measured under identical experimental conditions in the presence of the TMB/H_2_O_2_ substrate system. The inset shows representative amperometric current–time responses after substrate addition, illustrating the enhanced catalytic activity of the modified electrodes. Error bars represent the standard deviation of three independent measurements (*n* = 3). The improved response is attributed to the synergistic combination of V_2_O_5_ as the peroxidase-mimetic phase, with MWCNTs as conductive support, and HRP as enzymatic amplifier.

Electrochemical studies using the V_2_O_5_/MWCNTs-HRP–Streptavidin bioconjugate further supported enhanced activity. Amperometric measurements showed higher reduction currents and improved stability compared to electrodes modified with MWCNTs, V_2_O_5_, or their unmodified composite, demonstrating the synergistic effect of the conductive nanotube network and enzymatic amplification (Figure 3).

In the final bioconjugate, signal enhancement is therefore attributed to the combined contribution of the peroxidase-like V_2_O_5_ component, the electron-transfer properties of the MWCNT scaffold, and the catalytic activity of HRP.

### 3.3. Optimization of Immunosensor Parameters

The preparation of the immunosensor was based on the covalent immobilization of capture antibodies onto chemically modified SPCEs, followed by sequential formation of a sandwich-type immunocomplex and signal labeling with the V_2_O_5_/MWCNTs-HRP-Strep nanoconjugate (Figure 1). Surface functionalization was achieved via electrochemical grafting of *p*-aminobenzoic acid diazonium salt, providing carboxyl groups that were subsequently activated with EDC/NHS chemistry to enable stable antibody attachment. Residual active sites were passivated with bovine serum albumin prior to antigen recognition. After each modification and incubation step, the electrodes were rinsed with PBS to remove unbound species and minimize nonspecific contributions to the measured signal.

**Figure 4:**
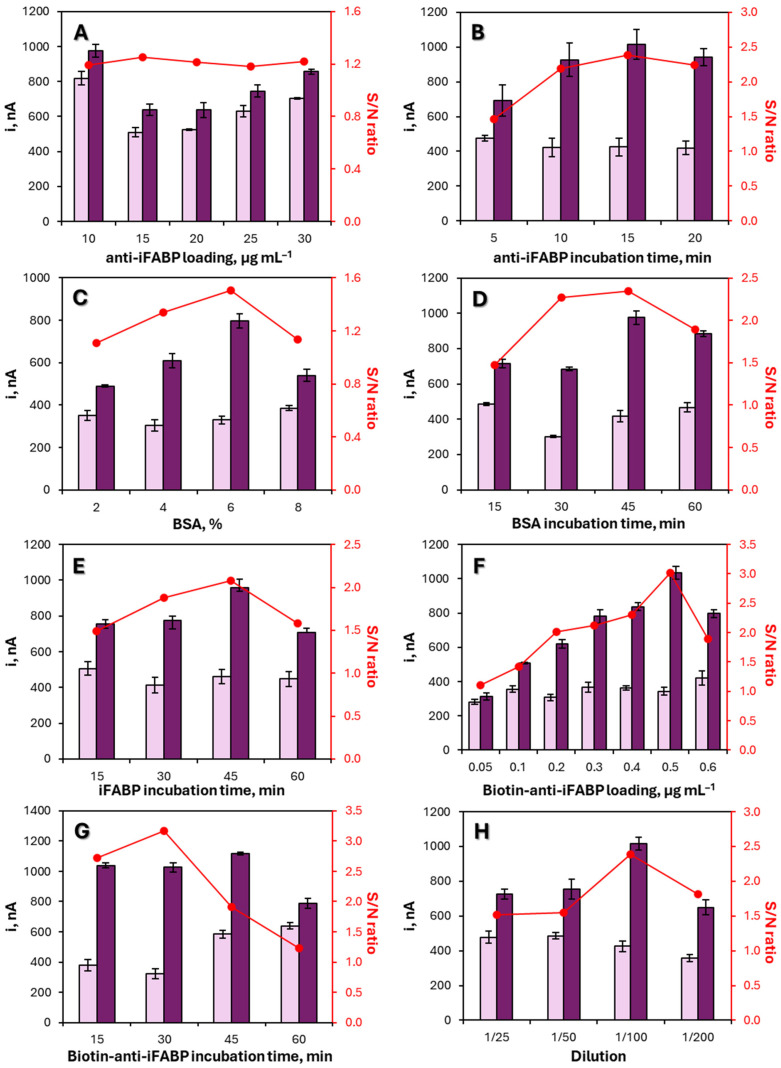
Optimization of the different experimental variables involved in the preparation of the electrochemical immunosensor for iFABP. Dependence of the amperometric responses measured in the absence (light purple, N) or in the presence (dark purple, S) of 1 ng mL^−1^ iFABP standards and the resulting signal-to-blank ratio (red lines, S/N) with the following: anti-iFABP concentration and incubation time (**A**,**B**); BSA concentration and incubation time (**C**,**D**); incubation time for iFABP standard (**E**); concentration of Biotin-anti-iFABP and incubation time (**F**,**G**); dilution of nanocomposite V_2_O_5_/MWCNTs/HRP-Strep (**H**). Unless otherwise specified, the concentration of iFABP used in the time-dependent panels (**B**,**D**,**E**,**G**) was fixed at 1 ng mL^−1^. The background signal (N) arises from the intrinsic electrochemical activity of the nanocomposite and nonspecific adsorption processes, as confirmed by control experiments in the absence of antigen. After each incubation step, electrodes were rinsed with PBS to remove unbound species and minimize nonspecific contributions. Incubation time = 20 min. Error bars estimated as triple of the standard deviation of three replicates.

To ensure reliable analytical performance, critical parameters affecting surface coverage, immunocomplex formation, and signal generation were systematically examined. Experimental conditions were selected by considering both the magnitude of the amperometric response and the discrimination between specific and nonspecific signals using 1 ng·mL^−1^ antigen as reference concentration. The background signal (N), measured in the absence of antigen, arises from the intrinsic electrochemical activity of the nanocomposite and nonspecific adsorption processes, and was therefore taken into account through the signal-to-background ratio (S/N). Optimization data for iFABP are presented in Figure 4, while those for FC are included in Figure S3. Control experiments corresponding to the background signal (N) are included to distinguish specific (S) and nonspecific contributions. The final selected conditions are summarized in Table 1.

The surface density of the capture antibody markedly influenced the analytical response. An intermediate concentration (10 μg·mL^−1^) provided balanced surface coverage, whereas lower loadings led to increased background currents (N) and higher concentrations negatively affected signal development, likely due to restricted accessibility of the target antigen. An immobilization period of 30 min was sufficient to ensure stable antibody attachment without additional improvement at longer times.

Blocking conditions were adjusted to minimize nonspecific adsorption. A BSA concentration of 3% (*w*/*v*) and an incubation time of 30 min provided effective surface passivation. Under these conditions, the background signal (N) was reduced and remained stable and reproducible. Under these conditions, antigen incubation times longer than 15 min did not enhance the analytical signal and were therefore not further considered.

Formation of the sandwich immunocomplex was optimized by evaluating the concentration and incubation time of the biotinylated detection antibody. A concentration of 0.5 μg mL^−1^ with a 30 min incubation ensured efficient complex formation while maintaining low background response (low N values and high S/N ratio).

Finally, the composition of the nanostructured enzymatic label was examined. Comparative experiments demonstrated that incorporation of V_2_O_5_ within the MWCNT framework significantly improved the electrochemical response relative to HRP-Strep alone or MWCNT-based conjugates. This enhanced response is associated with the catalytic contribution of the V_2_O_5_/MWCNT nanocomposite, which also contributes to the measurable background signal (N) observed in control experiments. The optimal proportion between HRP-Strep and V_2_O_5_/MWCNTs was achieved using 1 μL of enzyme conjugate per mL of nanocomposite. Subsequent dilution studies indicated that a 1/10 dilution provided the most favorable analytical performance and was selected for further measurements (Figure S4).

### 3.4. Electrochemical Characterization of the Developed Immunosensor

**Figure 5:**
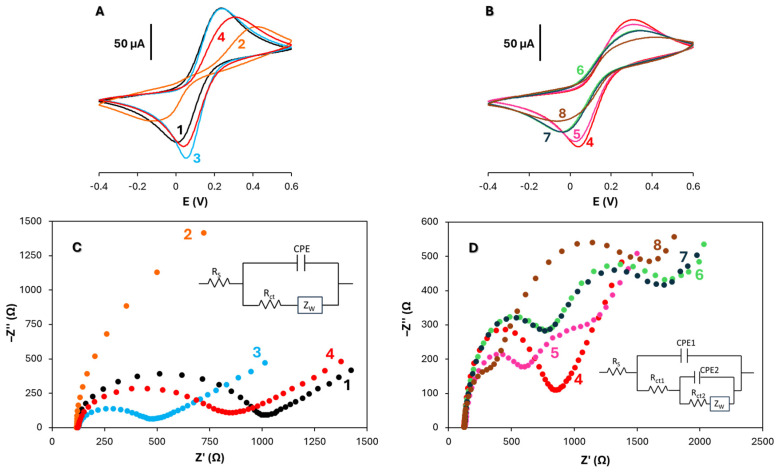
Cyclic voltammograms (**A**,**B**) and Nyquist plots (**C**,**D**) recorded for 5 mM Fe(CN)_6_^3−/4−^ in 0.1 mol L^−1^ PBS of pH 7.4 (scan rate 50 mV·s^−1^) at the following: (**A**,**C**) SPCE (1); HOOC-Phe-SPCE (2); HOOC-Phe-SPCE after EDC/sulfo-NHS activation (3); anti-iFABP-SPCE (4). (**B**,**D**) blocked anti-iFABP-SPCE (5); iFABP-anti-iFABP-SPCE (6); Biotin-anti-iFABP-iFABP-anti-iFABP-SPCE (7); V_2_O_5_/MWCNT-HRP-Strept-Biotin-anti-iFABP-iFABP-anti-iFABP-SPCE (8). The progressive modification of the electrode surface leads to changes in the electrochemical response, characterized by a decrease in peak currents in CV and an increase in charge transfer resistance (Rct) in EIS, confirming the stepwise assembly of the immunosensor. The equivalent circuits used to adjust the experimental results are shown within the figure, including Rs (solution resistance), Rct (charge transfer resistance), CPE (constant phase element), and Zw (Warburg impedance), allowing accurate fitting of the impedance data.

The stepwise construction of the dual immunosensor was assessed by cyclic voltammetry (CV) and electrochemical impedance spectroscopy (EIS) using 5 mM [Fe(CN)_6_]^3−/4−^ in 0.1 M PBS (pH 7.4) as redox probe (scan rate 50 mV·s^−1^). Comparable trends were observed for both target biomarkers; therefore, only the results obtained for iFABP are presented in Figure 5A–D. As the electrode modification progresses, a gradual decrease in peak currents in the cyclic voltammograms and a concomitant increase in charge transfer resistance (Rct) in the Nyquist plots are observed, reflecting the formation of an increasingly insulating biomolecular layer at the electrode surface. This behavior is consistent with the progressive hindrance of electron transfer caused by biomolecular layer formation.

Electrode modification with pABA diazonium salt led to a marked decrease in CV peak currents, which can be attributed to electrostatic repulsion between the negatively charged redox probe and the deprotonated carboxyl groups on the surface at neutral pH. Subsequent activation with EDC/sulfo-NHS partially restored peak reversibility, likely due to neutralization of the surface charges, producing a voltammogram closely resembling that of the bare SPCE. Immobilization of the anti-iFABP capture antibody reduced peak currents further, reflecting the insulating nature of the protein layer. This decrease in peak current is attributed to the increasing surface coverage and blocking effect induced by the immobilization of biomolecules, which limits the accessibility of the redox probe to the electrode surface. The blocking step with BSA produced a modest additional decrease, consistent with surface passivation. Incorporation of the remaining immunoreagents caused minor CV changes, indicative of the progressive formation of insulating layers of increasing thickness. Finally, deposition of the V_2_O_5_/MWCNTs/HRP-Strep nanocomposite decreased the currents further, likely as a result of the vanadium oxide component, despite the high conductivity of the MWCNT network.

EIS measurements corroborated the CV findings. Nyquist plots and equivalent circuit fittings are shown in Figure 5C,D. Grafting with *p*ABA increased the charge transfer resistance (Rct) from 900 Ω (bare SPCE) to 4377 Ω, reflecting hindered electron transfer. After EDC/sulfo-NHS activation, Rct decreased to 328 Ω, consistent with surface charge neutralization. Antibody immobilization increased Rct to 532 Ω, accompanied by the emergence of two semicircles in the EIS spectrum. The dual semicircle pattern indicates the presence of distinct interfacial processes: the smaller semicircle reflects solution/double-layer effects, while the larger semicircle represents specific charge transfer resistance associated with biomolecule adsorption or recognition events [21]. The diameter of the semicircle increased after each modification step, indicating a progressive increase in Rct due to the formation of insulating biomolecular layers on the electrode surface.

The Nyquist plots were fitted using two different equivalent circuits (Figure 5C, inset). Curves 1–4 were effectively described by a simple Randles-type circuit R1(C2[R3W1]). For curves 5–8, a more complex circuit with two RC semicircles was required, representing partial surface coverage by biomolecules alongside exposed electrode regions. Successive addition of BSA (curve 5), iFABP (curve 6), biotinylated detection antibody (curve 7), and V_2_O_5_/MWCNTs/HRP-Strep nanoconjugate (curve 8) led to progressive increases in the second semicircle diameter, with corresponding Rct values of 1179 Ω, 1411 Ω, 1554 Ω, and 1750 Ω, respectively, reflecting the insulating contribution of each layer (Figure 5D). The equivalent circuit includes Rs (solution resistance), Rct (charge transfer resistance), CPE (constant phase element), and Zw (Warburg impedance), allowing accurate fitting of the impedance data.

Overall, the electrochemical data confirms successful stepwise assembly of the immunosensor, with each modification producing predictable changes in both electron transfer kinetics and interfacial impedance, consistent with effective biomolecule immobilization and formation of the final nanostructured immunocomplex.

### 3.5. Analytical Characteristics of the Dual Immunosensor for the Simultaneous Determination of iFABP and FC Biomarkers

**Figure 6:**
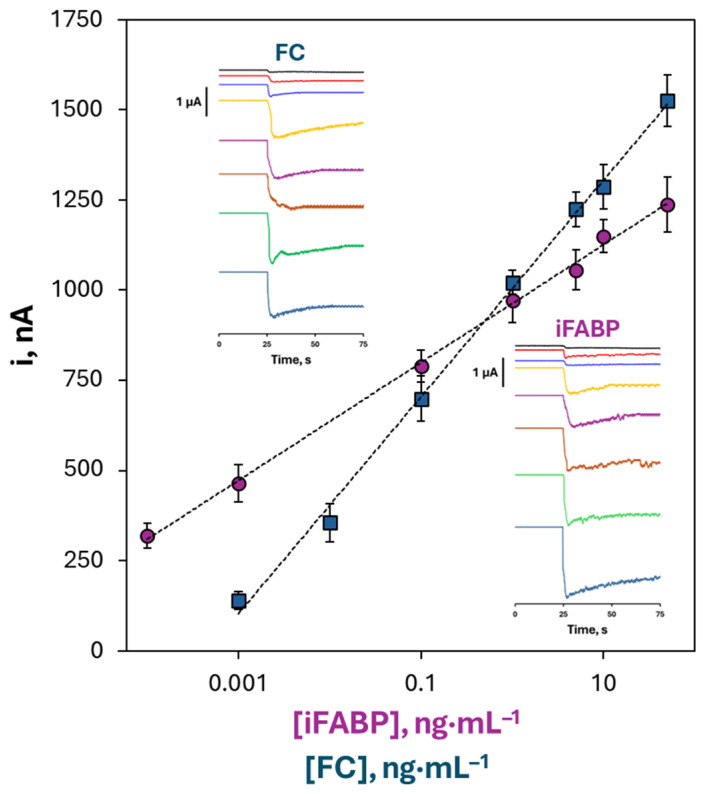
Calibration plot constructed with the developed dual immunosensor for the amperometric determination of iFABP and FC standards in the concentration range studied under optimized experimental conditions. The plots represent the steady-state current responses (i, nA) obtained after addition of H_2_O_2_, showing the simultaneous and independent detection of both biomarkers. Error bars are estimated as triple of the standard deviation of three replicates. Insets show representative chronoamperometric responses for increasing concentrations of FC (top) and iFABP (bottom), illustrating the stepwise increase in signal with analyte concentration.

The dual immunosensor was evaluated under the optimized conditions for the simultaneous quantification of iFABP and FC. Calibration plots obtained from amperometric measurements are shown in Figure 6, with representative amperograms. In accordance with the sandwich immunoassay design, the recorded currents increased with biomarker concentration. The analytical signal (S) was defined as the current measured in the presence of antigen, while the background signal (N) corresponds to measurements in its absence. Semilogarithmic calibration curves exhibited wide linear ranges, spanning 0.0001–100 ng mL^−1^ for iFABP and 0.001–100 ng mL^−1^ for FC. Key analytical parameters derived from these curves are summarized in Table 2. Limits of detection (LOD) and quantification (LOQ) were calculated using the *3s_b_*/m and *10s_b_*/m criteria, where *s_b_* represents the standard deviation of the background signal (N) obtained from ten measurements in the absence of target protein and *m* is the slope of the respective calibration curve.

Reproducibility was assessed by measuring 1 ng·mL^−1^ standards with ten independently prepared immunoconjugates using V_2_O_5_/MWCNTs/HRP-Strep, both within the same day (RSD = 4.2% for iFABP, 4.0% for FC) and across different days (RSD = 4.0% for iFABP, 4.1% for FC). Despite the presence of a measurable background signal (N), its stability ensured consistent signal-to-background ratios (S/N), supporting reliable quantification. These results indicate consistent sensor fabrication and stable electrochemical responses.

Compared to commercially available ELISA kits employing the same immunoreagents, the dual immunosensor offers several advantages. While ELISA calibration curves were non-linear and logarithmic, with narrower dynamic ranges (31.2–2000 pg mL^−1^ for iFABP and 93.8–6000 pg mL^−1^ for FC), the developed platform provided broader linear ranges and improved sensitivity. The use of S/N as analytical criterion minimizes the influence of the background signal, allowing accurate comparison across experimental conditions. The assay time was significantly reduced (1 h 20 min versus 4 h 40 min from BSA blocking to final measurement), and the required sample volume was minimal (5 μL versus 100 μL), making it particularly advantageous when sample availability is limited or multiple determinations are needed.

The combination of rapid analysis, low sample consumption, simple assay protocols, and portable, cost-effective instrumentation underscores the potential of this dual immunosensor for point-of-care testing (POCT), enabling fast and reliable assessment of clinically relevant biomarkers for gut inflammation monitoring.

### 3.6. Storage Stability and Selectivity

The long-term stability of the prepared immunosensing platforms (anti-iFABP–Phe-SPCEs and anti-FC-Phe-SPCEs) was assessed by constructing multiple sensors on the same day and storing them dry at 4 °C. The stored sensors were subsequently used to measure 10 ng mL^−1^ of each target biomarker over a 40-day period. Figure S5 presents a control chart in which the average current from ten sensors prepared on the first day was set as the central reference, with control limits defined as ±3 × s_b_. The measured amperometric responses remained within these limits throughout the storage period, demonstrating the excellent stability of the immobilized immunoconjugates.

**Figure 7:**
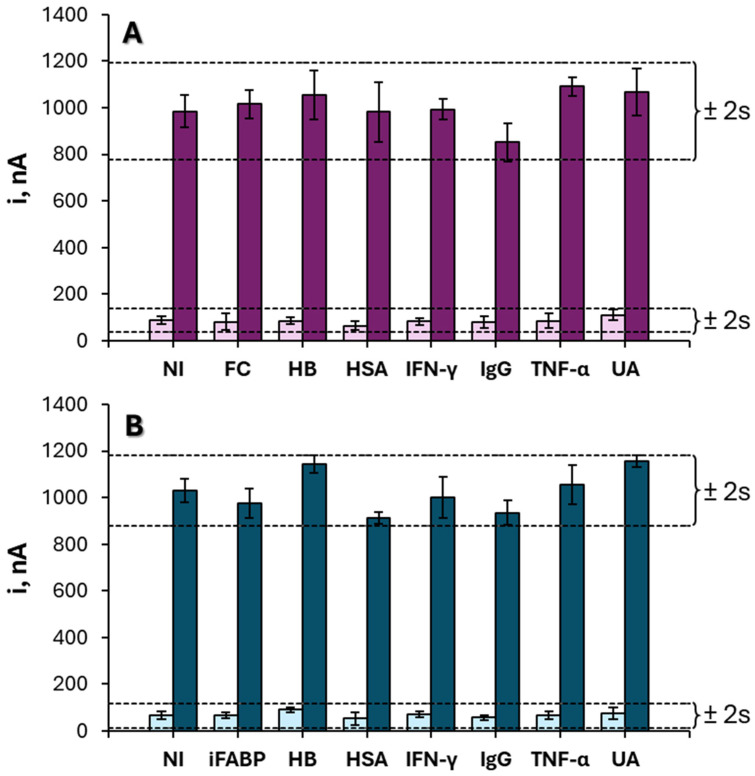
Amperometric responses provided by the developed immunosensor for the following: (**A**) 0 (light pink) and 10 ng mL^−1^ (dark pink) iFABP or (**B**) 0 (light blue) and 10 ng mL^−1^ (dark blue) FC in the presence of the following non-target compounds: 10 ng mL^−1^ FC (**A**) or iFABP (**B**), 5 mg mL^−1^ hemoglobin (HB), 50 mg mL^−1^ human serum albumin (HSA), 100 pg mL^−1^ interferon gamma (INF-γ), 1 mg mL^−1^ human immunoglobulin G(IgG), 200 pg mL^−1^ tumoral necrosis factor alpha (TNF-α) and 100 μg mL^−1^ uric acid (UA). The dashed lines represent the mean signal ±2 standard deviations obtained in the absence of interferents, providing a reference for evaluating potential interference effects. The results demonstrate that the presence of non-target species does not significantly affect the analytical signal, confirming the high selectivity of the immunosensor.

Selectivity was evaluated by examining the potential influence of non-target species on the sensor response. Amperometric measurements were performed for 0 and 10 ng mL^−1^ of iFABP or FC in the presence of potential interferents, including 1 ng mL^−1^ of the alternate biomarker, 5 mg mL^−1^ hemoglobin (HB), 50 mg mL^−1^ human serum albumin (HSA), 100 pg mL^−1^ interferon gamma (INF-γ), 1 mg mL^−1^ human immunoglobulin G (IgG), 200 pg mL^−1^ tumor necrosis factor alpha (TNF-α), and 100 μg mL^−1^ uric acid (UA). As shown in Figure 7, the average steady-state currents remained within ±3 standard deviations of the values recorded in the absence of interferents, indicating that the immunosensors maintain high specificity toward their respective targets (Figure 7).

Potential cross-reactivity between iFABP and FC was also assessed at 1 ng mL^−1^ by measuring amperometric responses for different mixtures of the two biomarkers (Figure S6). No significant interference was observed between the two electrodes, confirming the suitability of the dual-platform configuration for simultaneous and independent quantification of both analytes. Importantly, these results confirm the absence of significant cross-interference between both sensing channels under the tested conditions.

### 3.7. Analysis of Enriched Fecal Samples

The dual immunosensor was applied to the simultaneous quantification of iFABP and FC in human fecal samples fortified with 50 and 200 pg·mL^−1^ of each biomarker. These concentration levels were selected to represent clinically relevant conditions, covering low and moderately elevated biomarker levels associated with intestinal damage and inflammation. Although fecal calprotectin is commonly expressed in µg g^−1^, the concentrations used here correspond to diluted sample extracts and are consistent with clinically relevant levels when considering the sample preparation procedure.

Fecal samples were collected using an Eiken OC-Auto Sampling Bottle 3 (Ref. V-PZ25) and dispersed in 2 mL of HEPES buffer per 10 mg of sample. For each enriched level, five independent samples (*n* = 5) were analyzed to evaluate reproducibility and robustness. All sample handling and measurements were conducted in accordance with institutional ethical guidelines and relevant regulations.

Recovery studies demonstrated that the immunosensor reliably quantified both biomarkers in real fecal matrices, with recoveries ranging from 91.5% to 100.3% at the two enriched levels tested (Table 3). These results confirm the accuracy and reproducibility of the developed platform for direct analysis of complex biological samples, highlighting its potential for non-invasive monitoring of gut-related biomarkers.

## 4. Discussion

The V_2_O_5_/MWCNT hybrid nanocomposite significantly enhances immunosensor performance. Morphological and spectroscopic analyses confirmed uniform integration of V_2_O_5_ flakes onto the conductive nanotube network. Structural reorganization of V_2_O_5_, evidenced by Raman spectroscopy, promotes electron transfer and preserves redox-active sites, establishing a clear structure–function relationship.

Peroxidase-mimetic activity, combined with HRP enzymatic amplification, produced a synergistic enhancement of the amperometric signal. CV and EIS confirmed controlled stepwise assembly and predictable electrochemical behavior. The improved response is attributed to the synergistic combination of V_2_O_5_ as the peroxidase-mimetic phase, MWCNTs as conductive support, and HRP as enzymatic amplifier.

Analytically, the platform exhibited wide linear ranges, low detection limits, excellent reproducibility, and strong selectivity. Stability studies indicated long-term sensor integrity, and recovery in fortified feces confirmed robustness in real samples. Importantly, the absence of significant cross-interference between the two sensing channels further supports the reliability of the platform for simultaneous detection of multiple biomarkers in complex matrices.

Overall, integrating redox-active oxide with conductive nanotubes and enzymatic labeling yields a nanostructured platform that improves sensitivity, reduces assay time, and allows low-volume, portable measurements. This strategy demonstrates the potential of hybrid nanozyme-based systems for advanced electrochemical immunosensing and point-of-care applications.

## 5. Conclusions

In this work, we successfully developed a dual electrochemical immunosensor capable of detecting iFABP and FC simultaneously with high sensitivity and specificity. The incorporation of V_2_O_5_/MWCNTs-HRP–streptavidin nanocomposites enhanced electrocatalytic reduction in hydrogen peroxide, providing a strong signal amplification. Careful optimization of capture antibody loading, blocking conditions, antigen incubation, and nanocomposite labeling ensured consistent and reproducible sensor assembly, as confirmed by CV and impedance spectroscopy.

The platform demonstrated robust analytical performance, including excellent reproducibility, selectivity, and operational stability. Compared to conventional ELISA kits, our sensor required minimal sample volumes and shorter assay times while delivering comparable accuracy. The use of disposable screen-printed electrodes and a straightforward assay protocol further improve practicality and cost-effectiveness.

Validation with human fecal samples spiked with clinically relevant levels of iFABP and FC confirmed accurate quantification and reliable performance in complex matrices.

Overall, this dual immunosensing approach provides a promising route for rapid, multiplexed assessment of intestinal barrier function and gut inflammation. Its compact design, fast response, and compatibility with portable electrochemical systems make it well-suited for point-of-care applications, offering a viable alternative to traditional laboratory-based assays.

## Figures and Tables

**Table 1:** Experimental variables tested and values selected for the simultaneous determination of iFABP and FC with the developed immunosensors.

Target Biomarker	Variable	Tested Range	Selected Value
**iFABP**	anti-iFABP loading, μg mL^−1^ anti-iFABP incubation time, min BSA concentration, % BSA blocking time, min iFABP incubation time, min Biotin-anti-iFABP loading, μg mL^−1^ Biotin-anti-iFABP incubation time, min HRP-Strept volume, µL V_2_O_5_/MWCNTs/HRP-Strep dilution V_2_O_5_/MWCNTs/HRP-Strep incubation time, min	10–30 5–20 2–8 15–60 15–60 0.05–0.6 15–60 0.5–2 1/25–1/200 10–30	15 15 6 45 45 0.5 30 1 1/100 20
**FC**	Anti-FC loading, μg mL^−1^ Anti-FC incubation time, min BSA concentration, % BSA blocking time, min FC incubation time, min Biotin-anti-FC loading, μg mL^−1^ Biotin-anti-FC incubation time, min HRP-Strept volume, µL V_2_O_5_/MWCNTs/HRP-Strep dilution V_2_O_5_/MWCNTs/HRP-Strep incubation time, min	2.5–20 15–45 1–6 15–45 15–45 0.25–0.75 15–60 0.5–2 Undiluted—1/20 10–30	10 30 3 30 15 0.5 30 1 1/10 20

**Table 2:** Analytical characteristics of the calibration plots for iFABP and FC constructed with the dual immunoplatform.

Parameter	iFABP	FC
Slope, nA	164 ± 3	301 ± 7
Intercept, nA	963 ± 7	1005 ± 12
Linear range	0.1–50,000 pg·mL^−1^	1–50,000 pg·mL^−1^
R	0.998	0.997
LOD	0.01 pg·mL^−1^	1 pg·mL^−1^
LOQ	0.13 pg·mL^−1^	4 pg·mL^−1^
RSD, % (*n* = 10, intra-day)	4.6 (0 ng·mL^−1^) 4.2 (1 ng·mL^−1^)	4.9 (0 ng·mL^−1^) 4.0 (1 ng·mL^−1^)
RSD, % (*n* = 10, inter-day)	4.7 (0 ng·mL^−1^) 4.0 (1 ng·mL^−1^)	4.5 (0 ng·mL^−1^) 4.1 (1 ng·mL^−1^)

**Table 3:** Simultaneous determination of iFABP and FC in fortified feces with the dual immunosensor.

Fecal Sample	iFABP Added, pg·mL^−1^	iFABP Found, pg·mL^−1^	Recovery, %
1	50	50 ± 12	100.3
2	200	197 ± 34	98.7
**Fecal sample**	**FC added, pg·mL^−1^**	**FC found, pg·mL^−1^**	**Recovery, %**
1	50	46 ± 7	91.5
2	200	196 ± 7	98.2

*n* = 5; α = 0.05.

## Data Availability

The data will be available on request.

## Supplementary Materials

The following supporting information can be downloaded at https://www.mdpi.com/article/10.3390/bios16040199/s1, Figure S1: Vibrational Raman spectra of MWCNTs, V_2_O_5_, and V_2_O_5_/MWCNTs showing the characteristic bands of each component and confirming the successful formation of the hybrid nanocomposite. The spectra of MWCNTs display the typical D (~1350 cm^−1^) and G (~1580 cm^−1^) bands associated with disordered and graphitic carbon structures, respectively, while V_2_O_5_ exhibits characteristic vibrational modes in the low-frequency region. The V_2_O_5_/MWCNTs spectrum combines features of both materials, indicating the coexistence of vanadium oxide and carbon nanotubes without significant structural disruption; Figure S2: Evaluation of the peroxidase-like activity of V_2_O_5_/MWCNTs-HRP-Strept through the catalytic oxidation of TMB in a ready-to-use TMB/H_2_O_2_ solution. The plot shows the absorbance obtained after the reaction as a function of the added volume of V_2_O_5_/MWCNTs-HRP-Strept dispersion (0–100 µL) under identical experimental conditions. The inset photographs illustrate the corresponding color development of the TMB reaction mixture: (A) TMB/H_2_O_2_ solution without catalyst; (B) 25 µL of V_2_O_5_/MWCNTs-HRP-Strept; (C) 50 µL of V_2_O_5_/MWCNTs-HRP-Strept; (D) 50 µL of V_2_O_5_/MWCNTs-HRP-Strept; and (E) 25 µL of V_2_O_5_ in the presence of TMB/ H_2_O_2_. All assays were carried out under identical reaction conditions to allow a consistent comparison of catalytic activity; Figure S3: Optimization of the different experimental variables involved in the preparation of the electrochemical immunosensor for FC. Dependence of the amperometric responses measured in the absence (light blue, N) or in the presence (dark blue, S) of 1 ng mL^−1^ FC standards and the resulting signal-to-blank ratio (red lines, S/N) with the following: anti-FC concentration and incubation time (A,B); BSA concentration and incubation time (C,D); incubation time for FC standard (E); concentration of Biotin-anti-FC and incubation time (F,G). Unless otherwise specified, the concentration of FC used in the time-dependent panels (B,D,E,G) was fixed at 1 ng mL^−1^. The background signal (N) arises from the intrinsic electrochemical activity of the nanocomposite and nonspecific adsorption processes, as confirmed by control experiments in the absence of antigen. After each incubation step, electrodes were rinsed with PBS to remove unbound species and minimize nonspecific contributions. Incubation time = 20 min. Error bars estimated as triple of the standard deviation of three replicates.; Figure S4: Optimization of the experimental conditions for the amperometric detection of FC. Bars represent the current responses measured in the absence of analyte (light blue bars, N) and in the presence of FC standards (middle and dark blue bars, S). The red lines indicate the corresponding signal-to-blank ratios (S/N). (A) Effect of the catalytic system: HRP-streptavidin (HRP-Strept), MWCNTs/HRP-Strept, and V_2_O_5_/MWCNTs/HRP-Strept nanocomposite. (B) Influence of the volume of HRP-Strept added to 1 mL of V_2_O_5_/MWCNTs dispersion. (C) Effect of the dilution of the V_2_O_5_/MWCNTs/HRP-Strept nanocomposite. Current responses were recorded after a 20 min incubation step. Error bars represent the standard deviation of three independent measurements (*n* = 3); Figure S5: Storage stability of blocked-anti-iFABP-Phe-SPCEs (A) or blocked-anti-FC-Phe-SPCEs (B) conjugates used to construct the immunosensor, evaluated over a 40-day period. Amperometric measurements were made in the absence and in the presence of 10 ng mL^−1^ iFABP and FC standards, respectively. The dashed lines represent the mean signal ±3 standard deviations, indicating the acceptable variability range of the response. Error bars are estimated as triple of the standard deviation of three replicates. The results demonstrate good storage stability of the modified electrodes, with no significant loss of signal over time; Figure S6: Simultaneous amperometric responses measured with the V_2_O_5_/MWCNT-HRP-Strept-Biotin-anti-iFABP-iFABP-anti-iFABP–Phe-SPdCE (W1, light purple) and V_2_O_5_/MWCNT-HRP-Strept-Biotin-anti-FC-FC-anti-FC-Phe-SPdCE (W2, light blue) biosensors corresponding to the selective detection of iFABP (W1) and FC (W2), respectively, for standard mixtures containing: (SPdCE1) 0 ng·mL^−1^ iFABP and 0 ng·mL^−1^ FC (background signal, N); (SPdCE2) 1 ng·mL^−1^ FC and 1 ng·mL^−1^ iFABP; (SPdCE3) 1 ng·mL^−1^ iFABP and 1 ng·mL^−1^ FC. These conditions allow evaluation of the independent response of each sensing channel in the absence and presence of the potential interferent biomarker. Error bars are estimated as triple the standard deviation of three replicates.
