# Supplementary material for: Nanocomposite-Based Dual Electrochemical Immunosensor for Simultaneous Detection of Intestinal Barrier Biomarkers: Intestinal Fatty Acid Binding Protein and Fecal Calprotectin
Source: Biosensors (Basel). 2026 Apr 1;16(4):199. doi: 10.3390/bios16040199 (PMC13114182; doi:10.3390/bios16040199)
Supplement: Supplementary file 1 [file biosensors-16-00199-s001.zip › biosensors-4201708-supplementary.pdf]

Supplementary Materials

Nanocomposite-Based Dual Electrochemical Immunosensor for Simultaneous Detection of Intestinal Barrier Biomarkers: Intestinal Fatty Acid Binding Protein and Fecal Calprotectin

Lorena García-Rodrigo †, Claudia Ramos-López †, Esther Sánchez-Tirado *, Lourdes Agüí and Araceli González-Cortés *

Department of Analytical Chemistry, Faculty of Chemical Sciences, Universidad Complutense of Madrid, 28040 Madrid, Spain; lorega05@ucm.es (L.G.-R.); claura07@ucm.es (C.R.-L.); malagui@ucm.es (L.A.)

* Correspondence: esther.sanchez@ucm.es (E.S.-T.); aracelig@ucm.es (A.G.-C.)

† These authors contributed equally to this work.

3.1. Morphological characterization studies

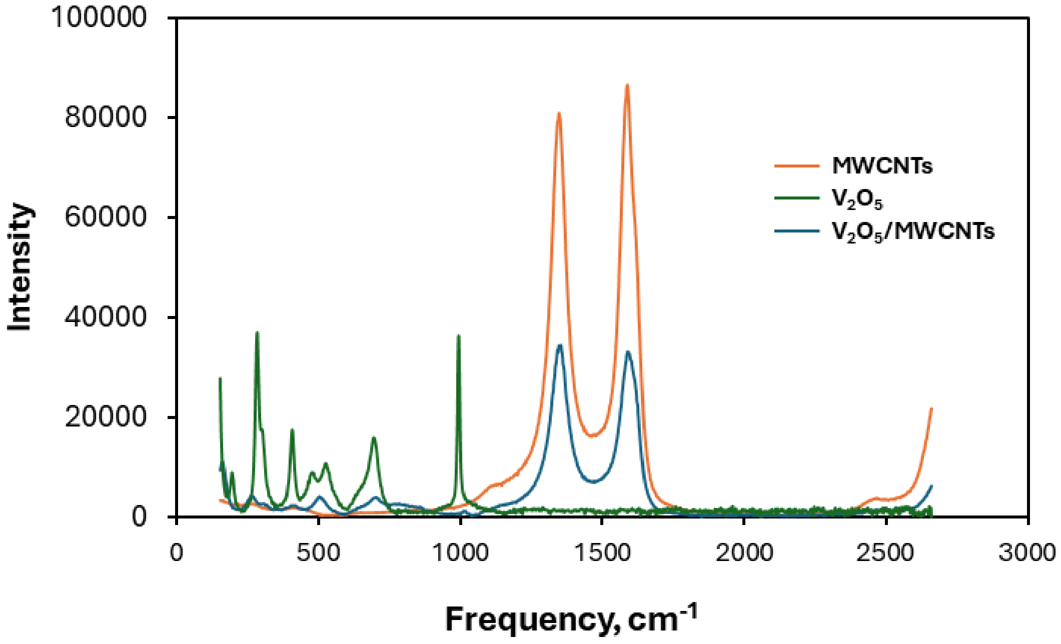

Figure S1. Vibrational Raman spectra of MWCNTs, V_2O_5, and V_2O_5/MWCNTs showing the characteristic bands of each component and confirming the successful formation of the hybrid nanocomposite. The spectra of MWCNTs display the typical D (~1350 cm^{-1}) and G (~1580 cm^{-1}) bands associated with disordered and graphitic carbon structures, respectively, while V_2O_5 exhibits characteristic vibrational modes in the low-frequency region. The V_2O_5/MWCNTs spectrum combines features of both materials, indicating the coexistence of vanadium oxide and carbon nanotubes without significant structural disruption.

3.2. Peroxidase-mimetic activity of V_2O_5/MWCNTs of nanocomposite

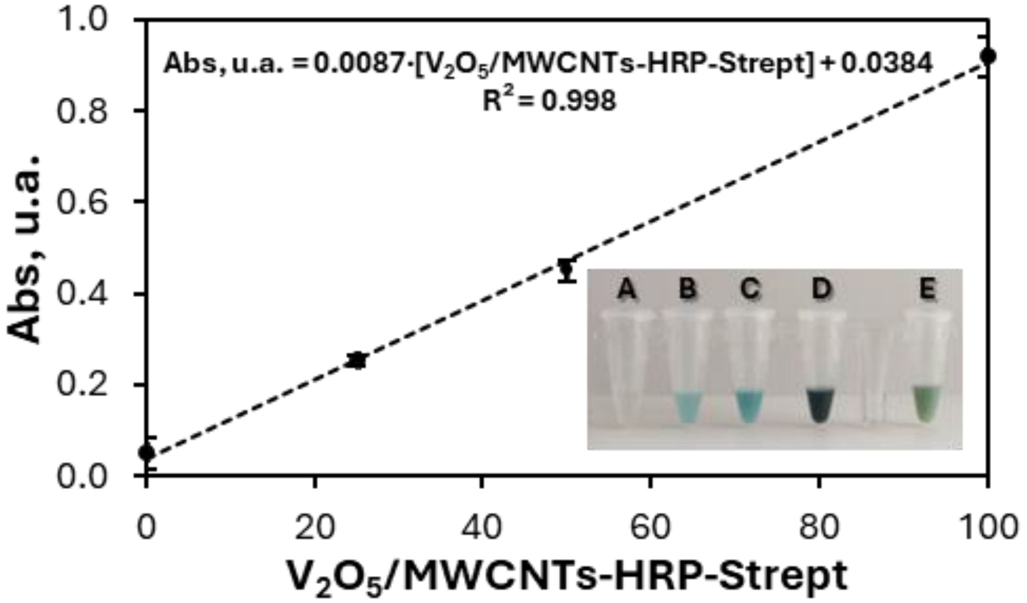

Figure S2. Evaluation of the peroxidase-like activity of V_2O_5/MWCNTs-HRP-Strept through the catalytic oxidation of TMB in a ready-to-use TMB/H_2O_2 solution. The plot shows the absorbance obtained after the reaction as a function of the added volume of V_2O_5/MWCNTs-HRP-Strept dispersion (0–100 μL) under identical experimental conditions. The inset photographs illustrate the corresponding color development of the TMB reaction mixture: (A) TMB/H_2O_2 solution without catalyst; (B) 25 μL of V_2O_5/MWCNTs-HRP-Strept; (C) 50 μL of V_2O_5/MWCNTs-HRP-Strept; (D) 50 μL of V_2O_5/MWCNTs-HRP-Strept; and (E) 25 μL of V_2O_5 in the presence of TMB/ H_2O_2. All assays were carried out under identical reaction conditions to allow a consistent comparison of catalytic activity.

3.3. Optimization of immunosensor parameters

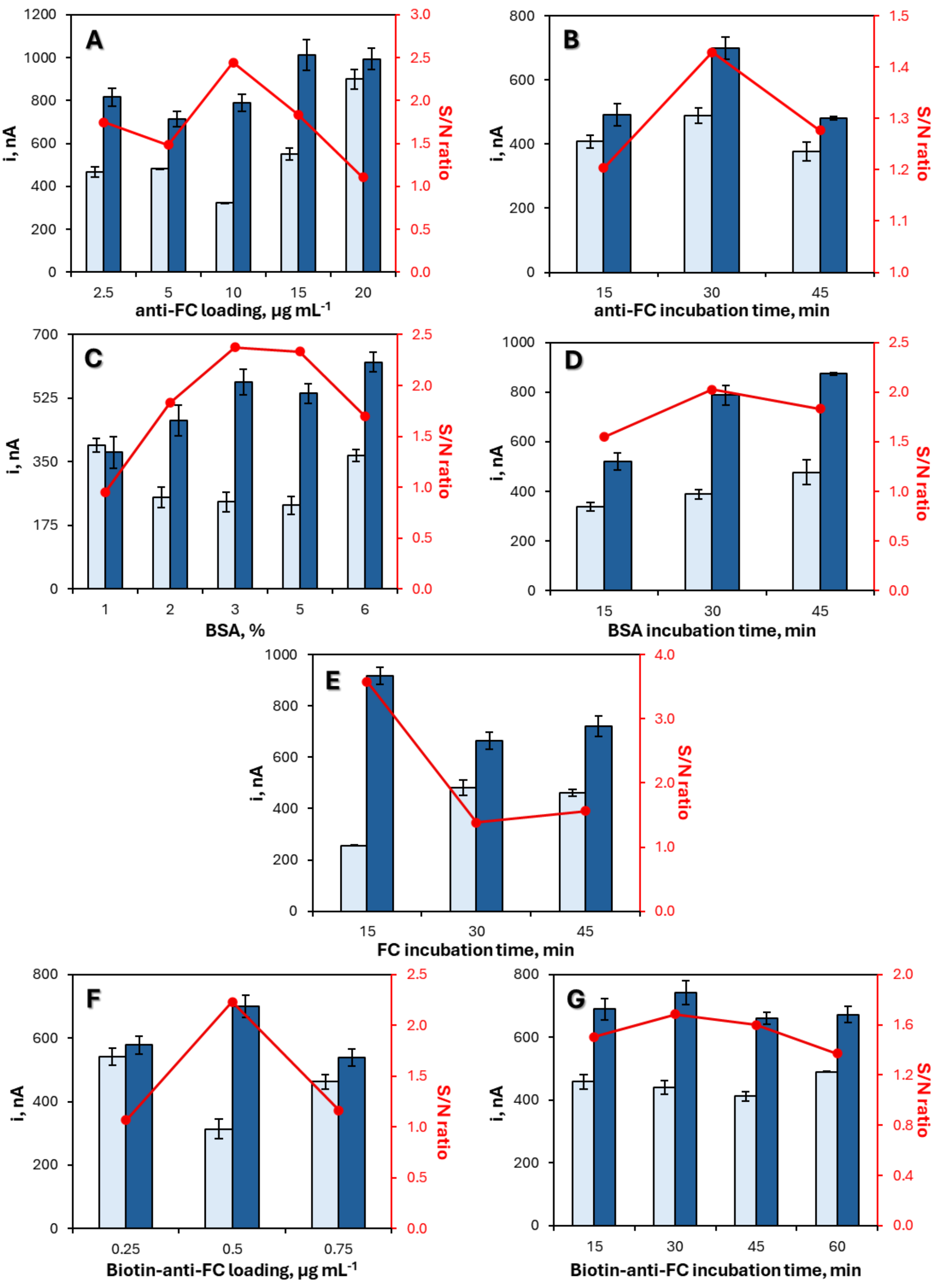

Figure S3. Optimization of the different experimental variables involved in the preparation of the electrochemical immunosensor for FC. Dependence of the amperometric responses measured in the absence (light blue, N) or in the presence (dark blue, S) of 1 ng mL⁻¹ FC standards and the resulting signal-to-blank ratio (red lines, S/N) with: anti-FC concentration and incubation time (A, B); BSA concentration and incubation time (C, D); incubation time for FC standard (E); concentration of Biotin-anti-FC and incubation time (F, G). Unless otherwise specified, the concentration of FC used in the time-dependent panels (B, D, E, and G) was fixed at 1 ng mL⁻¹. The background signal (N) arises from the intrinsic electrochemical activity of the nanocomposite and nonspecific adsorption processes, as confirmed by control experiments in the absence of antigen. After each incubation step, electrodes were rinsed with PBS to remove unbound species

and minimize nonspecific contributions. Incubation time = 20 min. Error bars estimated as triple of the standard deviation of three replicates.

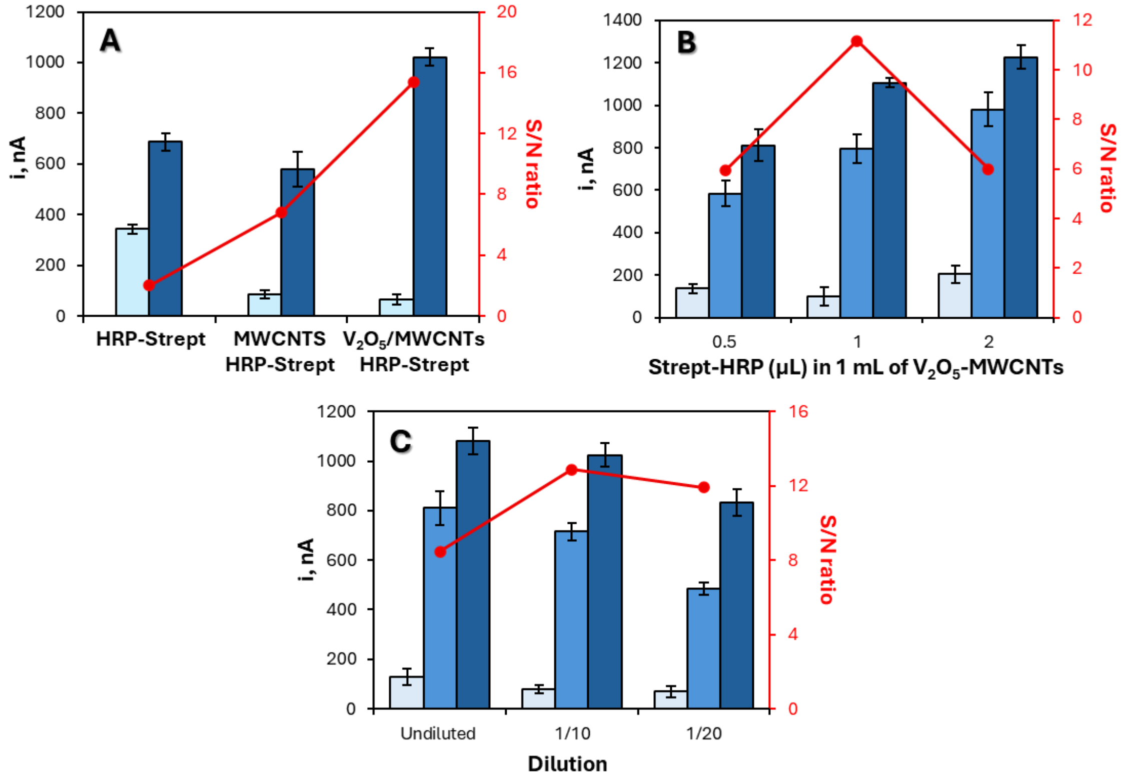

Figure S4. Optimization of the experimental conditions for the amperometric detection of FC. Bars represent the current responses measured in the absence of analyte (light blue bars, N) and in the presence of FC standards (middle and dark blue bars, S). The red lines indicate the corresponding signal-to-blank ratios (S/N). (A) Effect of the catalytic system: HRP-streptavidin (HRP-Strept), MWCNTs/HRP-Strept, and V_2O_5/MWCNTs/HRP-Strept nanocomposite. (B) Influence of the volume of HRP-Strept added to 1 mL of V_2O_5/MWCNTs dispersion. (C) Effect of the dilution of the V_2O_5/MWCNTs/HRP-Strept nanocomposite. Current responses were recorded after a 20 min incubation step. Error bars represent the standard deviation of three independent measurements (n = 3).

3.6. Storage stability and selectivity

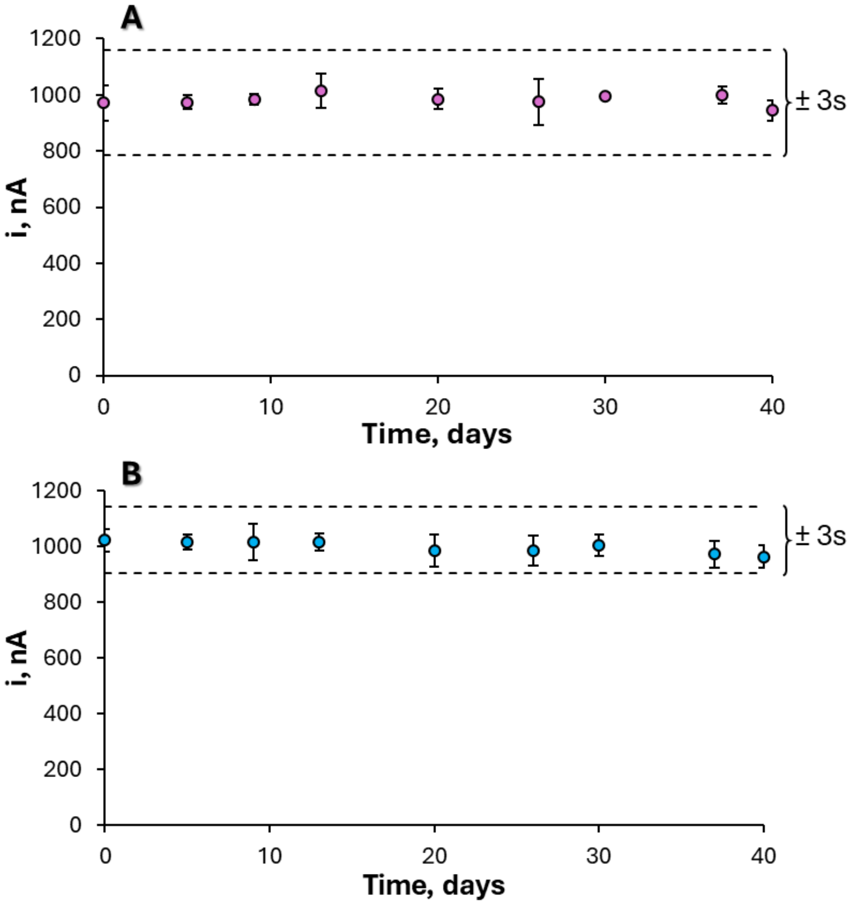

Figure S5. Storage stability of blocked-anti-iFABP-Phe-SPCEs (A) or blocked-anti-FC-Phe-SPCEs (B) conjugates used to construct the immunosensor, evaluated over a 40-day period. Amperometric measurements were made in the absence and in the presence of 10 ng mL^{-1} iFABP and FC standards, respectively. The dashed lines represent the mean signal ±3 standard deviations, indicating the acceptable variability range of the response. Error bars are estimated as triple of the standard deviation of three replicates. The results demonstrate good storage stability of the modified electrodes, with no significant loss of signal over time.

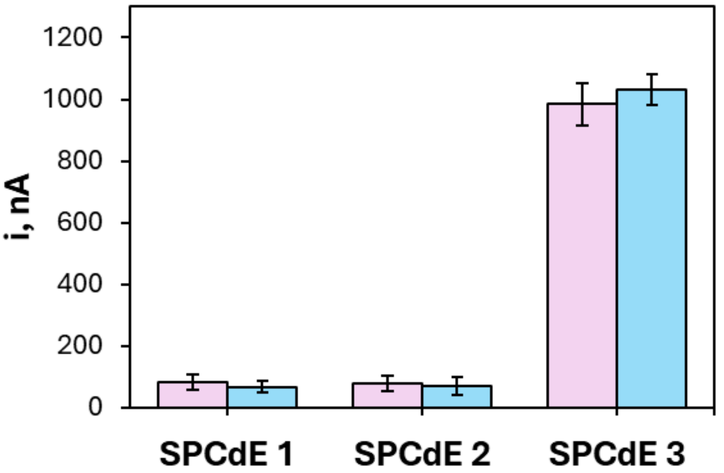

Figure S6. Simultaneous amperometric responses measured with the V_2O_5/MWCNT-HRP-Strept-Biotin-anti-iFABP-iFABP-anti-iFABP–Phe-SPdCE (W1, light purple) and V_2O_5/MWCNT -HRP-Strept-Biotin-anti-

FC-FC-anti-FC-Phe-SPdCE (W2, light blue) biosensors corresponding to the selective detection of iFABP (W1) and FC (W2), respectively, for standard mixtures containing: (SPdCE1) 0 ng·mL^{-1} iFABP and 0 ng·mL^{-1} FC (background signal, N); (SPdCE2) 1 ng·mL^{-1} FC and 1 ng·mL^{-1} iFABP; (SPdCE3) 1 ng·mL^{-1} iFABP and 1 ng·mL^{-1} FC. These conditions allow evaluation of the independent response of each sensing channel in the absence and presence of the potential interferent biomarker. Error bars are estimated as triple the standard deviation of three replicates.
